# Weakly-supervised learning for lung carcinoma classification using deep learning

**DOI:** 10.1038/s41598-020-66333-x

**Published:** 2020-06-09

**Authors:** Fahdi Kanavati, Gouji Toyokawa, Seiya Momosaki, Michael Rambeau, Yuka Kozuma, Fumihiro Shoji, Koji Yamazaki, Sadanori Takeo, Osamu Iizuka, Masayuki Tsuneki

**Affiliations:** 1 Medmain Research, Medmain Inc., Fukuoka, 810-0042 Japan; 2 Department of Thoracic Surgery, Clinical Research Institute, National Hospital Organization, Kyushu Medical Center, Fukuoka, 810-8563 Japan; 3 Department of Pathology, Clinical Research Institute, National Hospital Organization, Kyushu Medical Center, Fukuoka, 810-8563 Japan; 4 Medmain Inc., Fukuoka, 810-0042 Japan

**Keywords:** Cancer, Lung cancer, Machine learning

## Abstract

Lung cancer is one of the major causes of cancer-related deaths in many countries around the world, and its histopathological diagnosis is crucial for deciding on optimum treatment strategies. Recently, Artificial Intelligence (AI) deep learning models have been widely shown to be useful in various medical fields, particularly image and pathological diagnoses; however, AI models for the pathological diagnosis of pulmonary lesions that have been validated on large-scale test sets are yet to be seen. We trained a Convolution Neural Network (CNN) based on the EfficientNet-B3 architecture, using transfer learning and weakly-supervised learning, to predict carcinoma in Whole Slide Images (WSIs) using a training dataset of 3,554 WSIs. We obtained highly promising results for differentiating between lung carcinoma and non-neoplastic with high Receiver Operator Curve (ROC) area under the curves (AUCs) on four independent test sets (ROC AUCs of 0.975, 0.974, 0.988, and 0.981, respectively). Development and validation of algorithms such as ours are important initial steps in the development of software suites that could be adopted in routine pathological practices and potentially help reduce the burden on pathologists.

## Introduction

Lung cancer is one of the leading causes of cancer-related deaths in many countries around the world, and its histopathological diagnosis is crucial for deciding on optimum treatment strategies. According to global cancer statistics^1^, in 2018, there were 2,093,876 (11.6% of all sites) new cases and 1,761,007 (18.4% of all sites) deaths due to lung cancer. Microscopic histopathology remains the gold standard in diagnostic surgical pathology; however, the main limitation to morphological diagnosis is diagnostic variability among pathologists^2^. These problems may have a negative impact on the quality of the pathological diagnosis, which should be addressed urgently.

The progressive use and adoption of digitised Whole Slide Images (WSIs) has allowed the use of biomedical image analysis techniques from computer vision and machine learning to aid the pathologists in obtaining diagnoses and has led to the emergence of computational pathology as a field^3^. Over the past few years, deep learning has been shown to have successful applications in computational pathology^4–16^. Most notably, Coudray *et al*.^10^ trained a deep learning model to classify and predict mutations from non–small cell lung cancer histopathology. While Wei *et al*.^11^ trained a deep learning model to classify lung adenocarcinoma on surgical resection slides using 279 WSIs for training and only 143 WSIs for testing.

One of the primary challenges of computational pathology is the size of the WSI. A single image scanned at a magnification of x20 can have a height and width in tens of thousands of pixels, which makes it time-consuming to visually inspect in an exhaustive manner by a pathologist. Due to this, the application of deep learning models has required splitting the image into a set consisting of thousands of tiles and applying the classification model on each tile separately, and then later combining the result for the WSI. In addition, the application of deep learning requires an annotated dataset to use to train the models, with larger datasets typically achieving better performance. However, what this entails is that detailed cell-level WSI annotations by expert pathologists are required, making it extremely difficult to compile a large dataset, especially for WSIs consisting of surgical specimens.

There are two extremes when it comes to annotating a WSI: detailed cell-level annotations and slide-level diagnosis. The former is the most tedious and time consuming to obtain and is a requirement for fully-supervised machine learning, while the latter is the fastest to obtain, and typically readily available from diagnostic reports. Applying machine learning using only a dataset of slide-level diagnoses involves posing the problem within a weakly-supervised learning^17^ setting, where the slide-level diagnoses constitute weak labels. Based on the slide-level diagnosis label of a given WSI, the following assumption can be made about the labels of its tiles: if a WSI has cancer then at least one tile from the WSI must contain cancer cells, while if the WSI has no cancer, then none of the tiles will contain any cancer cells. This formulation is called Multiple Instance Learning^18^ (MIL) and has found many applications in machine learning^19–22^. The most notable application of MIL in computational pathology was done recently by Campanella *et al*.^15^ in which they used it to train on a dataset of 44,732 WSIs using only slide-level diagnoses as labels with impressive results obtained on test sets of prostate cancer, basal cell carcinoma, and breast cancer metastases.

**Figure 1:**
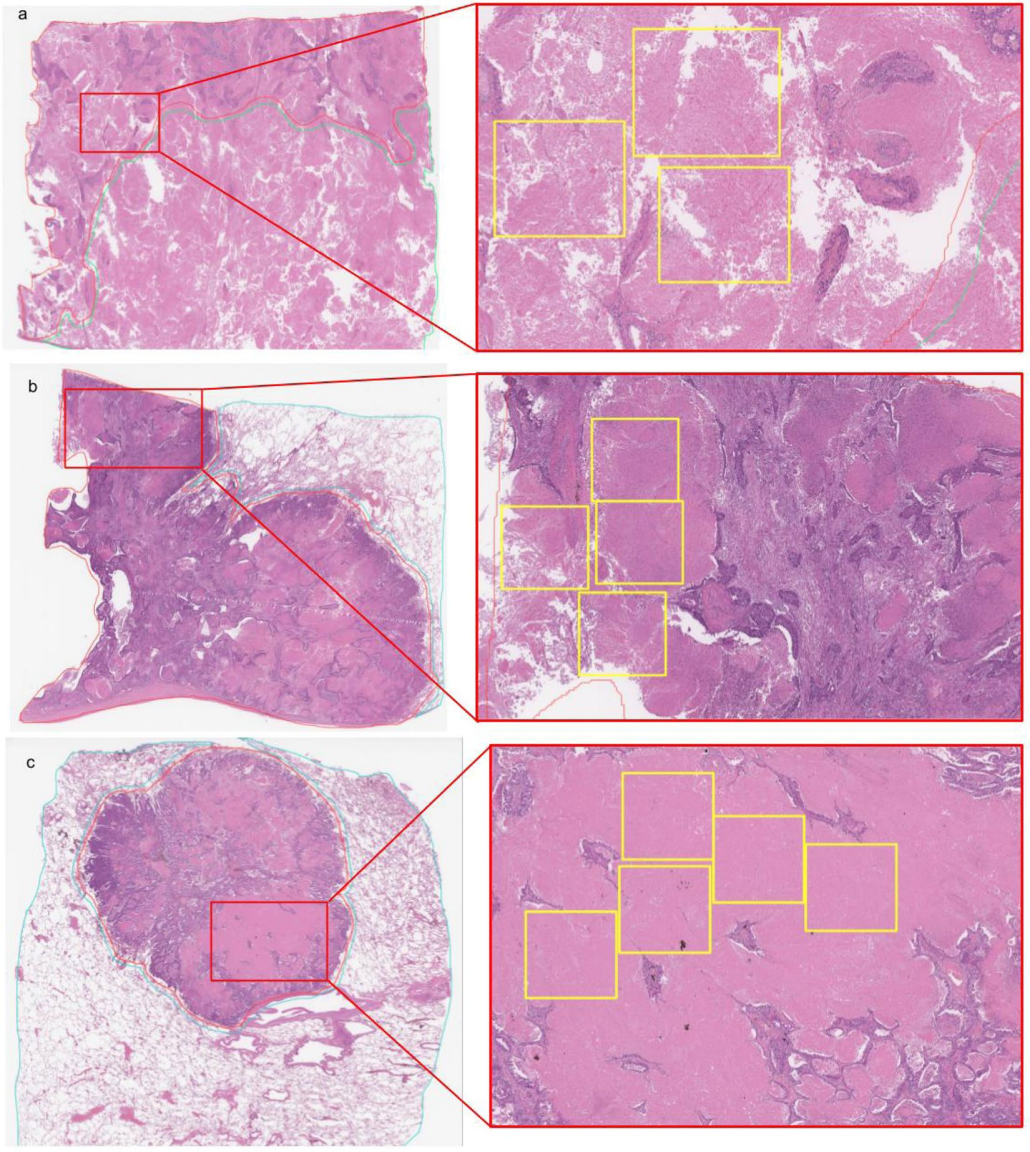
(**a**–**c**) Highlights showing three examples of carcinoma WSI annotations. The left column shows thumbnails of the WSIs, while the right shows zoomed-in crops from regions labelled as carcinoma. The yellow boxes indicate necrotic tissues within the tumour. Extracting tiles from tissue within yellow boxes would result in tiles consisting of necrotic tissue labelled as carcinoma. This would introduce noise within the dataset as necrotic tissue can also be part of non-neoplastic lesions.

Here, we propose a weakly-supervised deep learning method to predict carcinoma in WSIs of lung using a dataset annotated at a level in between the two extremes of annotations, where annotations were carried out but not at a cell-level detail. As our training dataset consisted of surgical specimens, one hurdle we encountered was the level of detail required to carry out the annotations. In some cases, cancer cells spread within necrotic or inflammatory tissues which are also present within purely non-neoplastic lesions. In addition, sometimes it is impossible to annotate carcinoma cells accurately because some undergo cell death due to chemotherapy treatment. If pathologists were to annotate at a cell-level detail it would take hours for a single WSI. Therefore, we opted for annotating the whole carcinoma areas without excluding the necrotic tissues within them. As a result of this, tiles extracted from carcinoma labelled areas could potentially originate from necrotic or inflammatory tissues which would also be similar to tissues in some tiles extracted from non-neoplastic lesions. Figure 1 illustrates examples of the annotations that we carried out. We did this to maximise the use of our collected training dataset which consisted of 3,704 WSIs of surgical lung specimens. We then used a combination of transfer learning and weakly-supervised learning to train an EfficientNet-B3^23^ Convolutional Neural Network (CNN). We evaluated our model on four independent test sets consisting of about 500 cases each. To the best of our knowledge, this makes our study the largest so far to evaluate on independent test sets for lung carcinoma classification.

## Results

### A deep learning model for WSI carcinoma classification

The purpose of this study was to train a deep learning model to classify carcinoma in WSIs and evaluate it on multiple independent cohorts. We used a dataset of 3,704 WSIs obtained from Kyushu Medical Centre. 3,554 WSI were used for training while 150 were used for validation. We then evaluated our model on five independent test sets: 500 from Kyushu Medical Centre, 500 from Mita Hospital, 670 from the publicly available repository of The Cancer Genome Atlas (TCGA) program^24^, and 500 from the Cancer Imaging Archive (TCIA).

Taking into account the nature of the annotations that we performed on the surgical specimens, we trained two models using the following two approaches: fully-supervised learning and weakly-supervised learning. The former typically performs best when detailed cell-level annotations are available. The latter approach can be used with weak labels, such as when only the slide-level diagnoses are available; however, it typically requires a much larger dataset of WSIs.

To obtain a WSI classification, the model was applied in a sliding window fashion with input tiles of 512 × 512 pixels and a stride of 256. The WSI classification was then obtained by taking the maximum probability of all its tiles.

**Figure 2:**
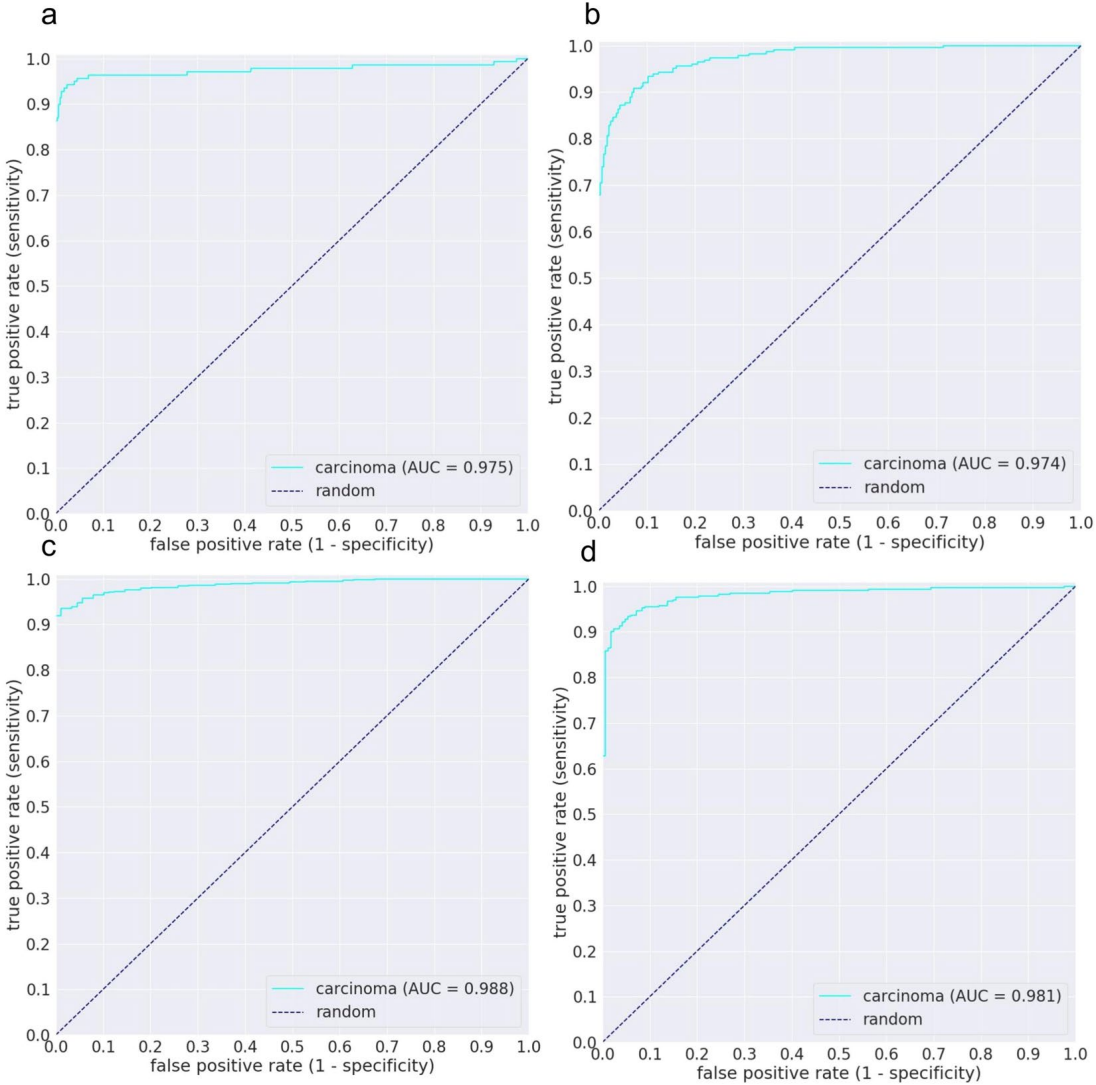
ROC curves of carcinoma WSI classification trained using the weakly-supervised MIL approach on four independent test sets: (**a**) Kyushu Medical Centre, (**b**) Mita Hospital (**c**) TCGA, and (**d**) TCIA.

**Figure 3:**
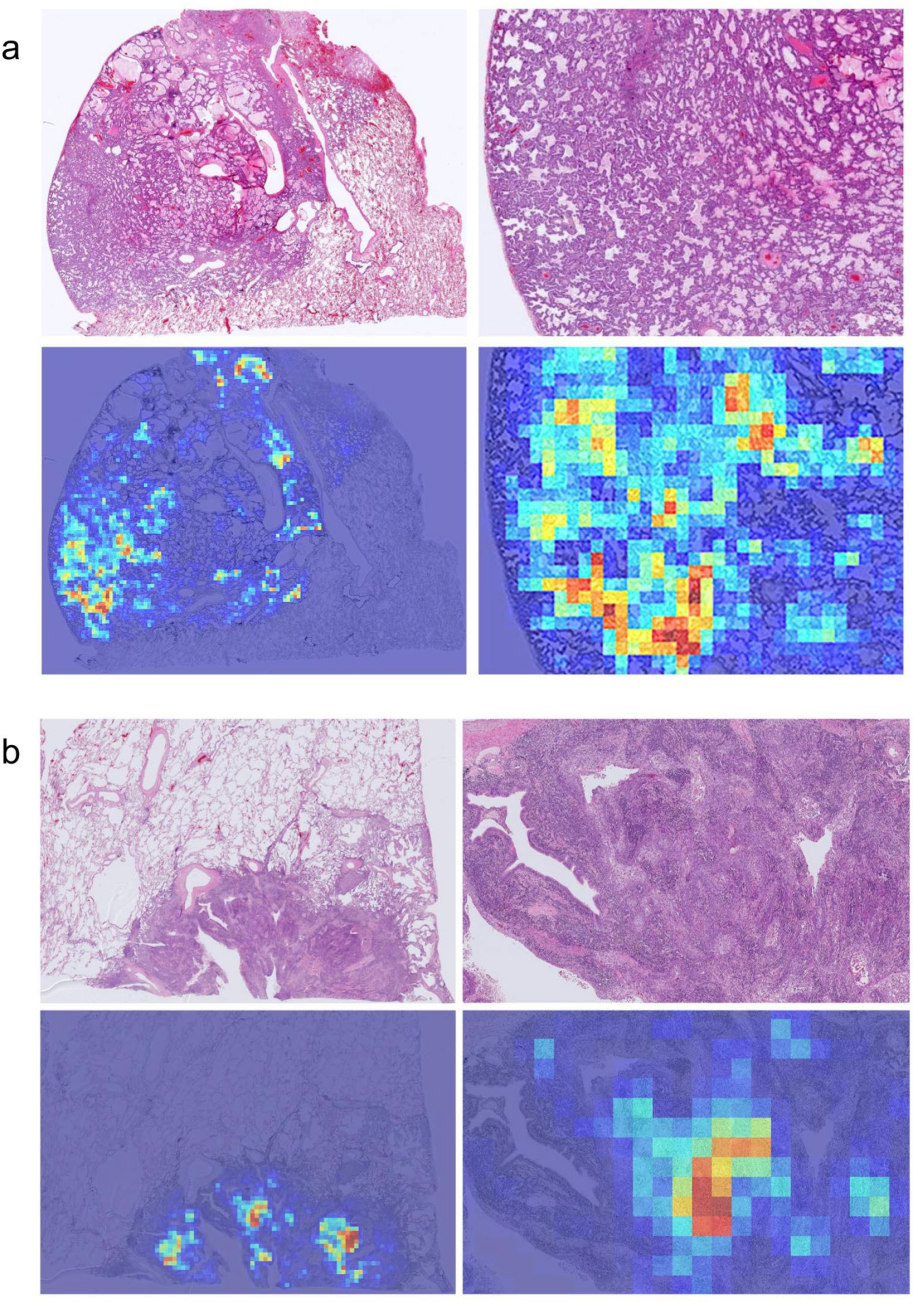
Example heatmap outputs for carcinoma prediction using the weakly-supervised method on (**a**) adenocarcinoma and (**b**) squamous cell carcinoma. A stride of 256 × 256 was used on a magnification of x10.

We then computed the ROC curves and their corresponding AUCs and the log losses. Figure 2 and Table 1 summarise the results on the four independent test sets using the two training methods. Figure 3 shows example heatmap output visualisations.

**Table 1:** Summary of ROC AUC and log loss results of the two training methods, fully-supervised (FS) and weakly-supervised (WS), on the 4 independent test sets: Kyushu Medical Centre, Mita Hospital, TCGA, and TCIA.

		Kyushu Medical Centre	Mita Hospital	TCGA	TCIA
WS	ROC AUC	0.975 (0.950–0.993)	0.974 (0.962–0.985)	0.988 (0.981–0.994)	0.981 (0.969–0.990)
	Log loss	0.1338	0.4045	0.1819	0.3506
FS	ROC AUC	0.937 (0.908–0.958)	0.922 (0.898–0.945)	0.880 (0.840–0.917)	0.963 (0.944–0.977)
	Log loss	0.7022	0.8138	0.2977	0.2813

The values between parenthesis indicate the 95% Confidence Intervals (CI)s.

## Discussion

In this work we trained a deep learning model for the classification of carcinoma in WSIs. The model was trained on WSIs obtained from a single medical institution and was then applied on four independent test sets obtained from different sources to demonstrate the generalisation of the model on unseen data.

We showed that it was possible to exploit the use of a moderately-size training dataset of 3,554 WSIs to train a deep learning model using a combination of transfer learning and weakly-supervised learning, and we have obtained high ROC AUC performance on four independent test sets, which is highly promising in terms of the generalisation performance of our model.

**Figure 4:**
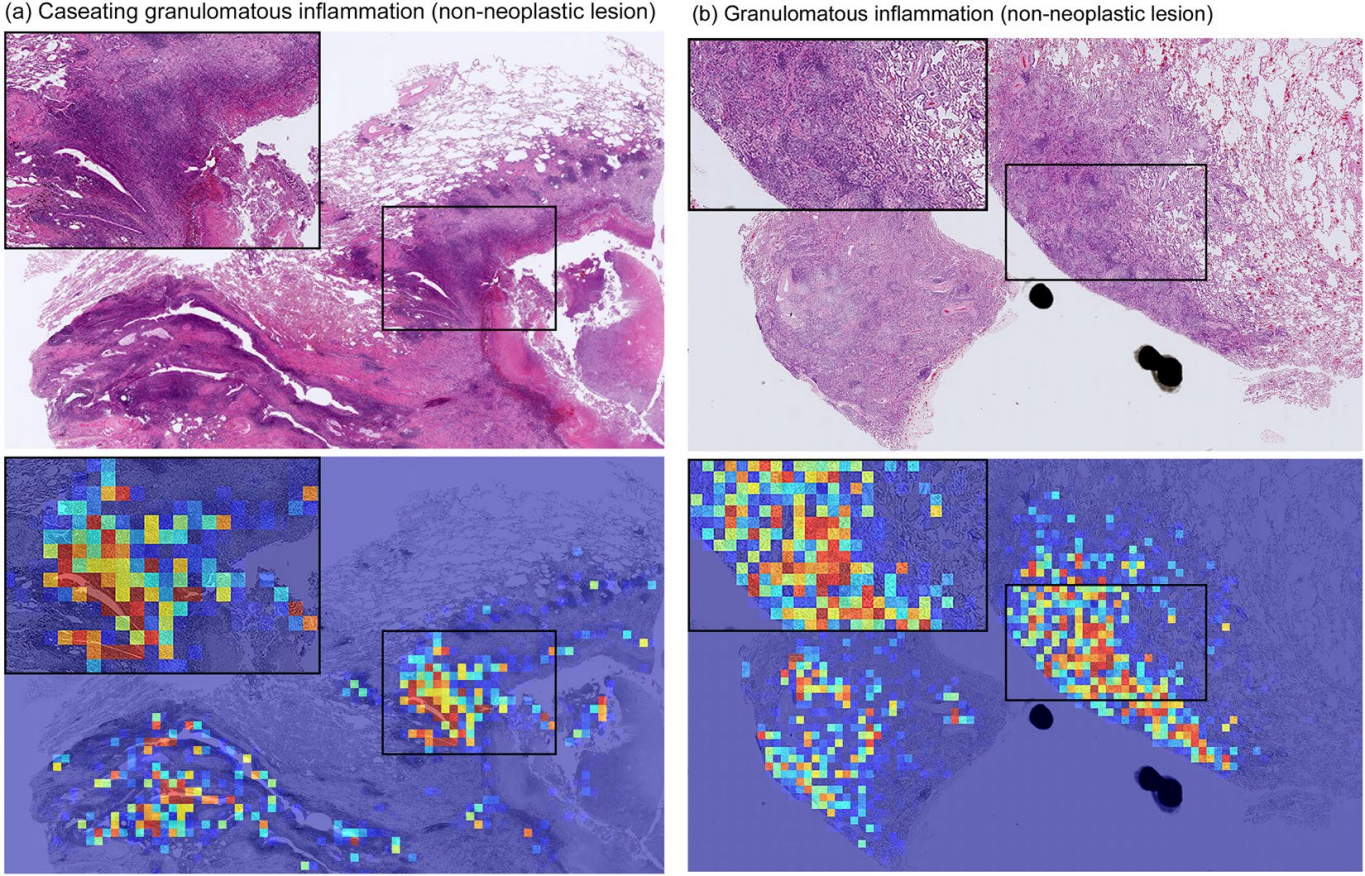
Examples of two carcinoma false positive prediction outputs using the fully-supervised method. Both were correctly predicted as non-neoplastic by the weakly-supervised method. (**a**) is a case of caseating granulomatous inflammation^36^ which consists of necrotic tissues (caseation necrosis is defined as a region in granulomas with eosinophilic, granular and cheese-like cellular debris with necrosis) and chronic inflammation. (**b**) is a case of granulomatous inflammation^37^, which is a distinctive form of chronic inflammation produced in response to various infection. The inflammatory tissue is the main cause of false positives due to its analogous tissue structure to cancer stroma.

We used two approaches to train the models: fully-supervised learning and weakly-supervised learning. The latter consistently performed statistically significantly better than the former, with an average improvement of about 0.05 in the AUC on the test sets. The TCGA set had the largest improvement in AUC of about 0.10; however, this could also be explained by the larger proportion of carcinoma cases vs non-neoplastic (591 vs 89) as compared to the other test sets, and therefore a reduction in the number of false-positives using the weakly-supervised method would lead to a larger improvement in AUC. One of the main issues of directly using fully-supervised learning was that due to the large size of the WSI surgical specimens, the annotations could not be carried at a detailed cell level as it would have taken a considerable amount of time to annotate a single WSI. As a result of the non-cell-level annotations, tiles extracted from regions labelled as carcinoma could originate from the stromata including necrotic or inflammatory tissues, which also could be shared with non-neoplastic lesions. This would result in such tiles either having a carcinoma label or a non-neoplastic label, depending on which labelled regions they originated from. This would introduce noise within the training set and result in a decrease in model performance, which is what we have observed using the fully-supervised learning method. Figure 4 illustrates two examples of false positive predictions by the the fully-supervised method on inflammatory tissues, which were correctly predicted as non-neoplastic by the weakly-supervised method. Using the weakly-supervised MIL method allowed us to train on our dataset and obtain high performance despite the non-cell level nature of the annotations. This means that it is possible to train a high performing model for lung carcinoma classification without having to have detailed cell-level annotations or requiring an extremely large number of WSI to perform pure MIL using only slide-level labels.

**Figure 5:**
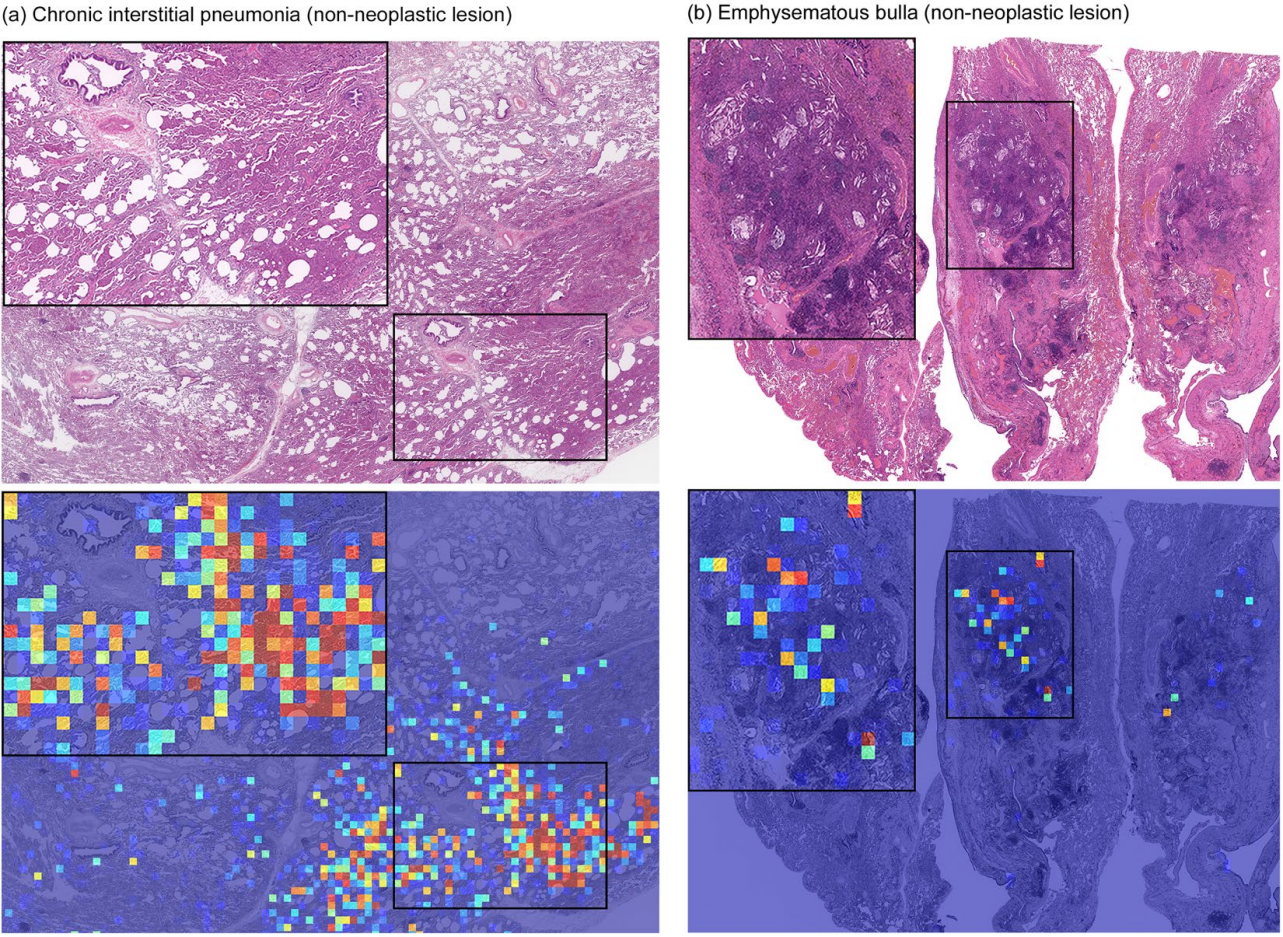
Examples of two carcinoma false positive prediction outputs using the weakly-supervised method. (**a**) Is a case of chronic interstitial pneumonia: there are prominent appearances of macrophages in the alveolar space and fibrosis of the alveolar septum as a background of mild to moderate chronic active inflammation. Infiltrating inflammatory cells include lymphocytes, monocytes, and neutrophils. (**b**) Is a case of emphysematous bulla: there is a cystic emphysematous space in the lung beneath the pleura. Chronic inflammation, congestion and hemorrhage are seen. The inflammatory tissues in (**a**,**b**) is the main cause of false positive prediction due to the resemblance to cancer stroma.

Using the weakly-supervised method, an inspection of false-negative cases showed that the majority were due to presence of only a few cancer cells in a small area, which might have made it difficult for the model to pick up at the chosen magnification or stride. Albeit a smaller number than that of the fully-supervised method, the majority of false-positives were due to false detections on inflammatory tissues. Figure 5 illustrates two examples of false positive carcinoma predictions using the weakly-supervised method.

Our model is currently limited to differentiating between carcinoma and non-neoplastic lesion. However, despite this limitation our model could be used as an initially applied algorithm in a workflow in which subsequently applied algorithms further subclassify neoplastic tissue and perform clinically useful functions, such as outlining lesional cells (which would be useful for detecting lymphovascular invasion), assessing pleural involvement (a feature that pathologists can struggle with) and assessing margin status. As future work, we are working on developing additional models to subclassify lung carcinoma, in surgical as well as biopsy specimens, into adenocarcinoma, squamous cell carcinoma, and small cell carcinoma; this subclassification is important for triaging tumors to receive appropriate molecular testing and to determine optimal therapy^25,26^.

## Methods

### Clinical cases

In the present retrospective study, 4,204 cases of human pulmonary lesions HE (hematoxylin & eosin) stained histopathological specimens were collected from the surgical pathology files of Kyushu Medical Center after histopathological review of those specimens by surgical pathologists. 500 cases of human pulmonary lesions HE stained surgical specimens were collected from International University of Health and Welfare, Mita Hospital (Tokyo) after histopathological review and approval by surgical pathologists. The experimental protocols were approved by the Institutional Review Board (IRB) of the Kyushu Medical Center (No. 19C106) and International University of Health and Welfare (No. 19-Im-007). All research activities comply with all relevant ethical regulations and were performed in accordance with relevant guidelines and regulations in Kyushu Medical Center and International University of Health and Welfare, Mita Hospital. Informed consent to use histopathological samples and pathological diagnostic reports for research purposes had previously been obtained from all patients prior to the surgical procedures at both hospitals and the opportunity for refusal to participate in research has been guaranteed by an opt-out manner. The test cases were selected randomly, so the ratio of neoplastic to non-neoplastic cases in test sets was reflective of the case distributions at the providing institutions. All WSIs from both Kyushu Medical Center and Mita were scanned at a magnification of x20.

### Dataset and annotations

**Figure 6:**
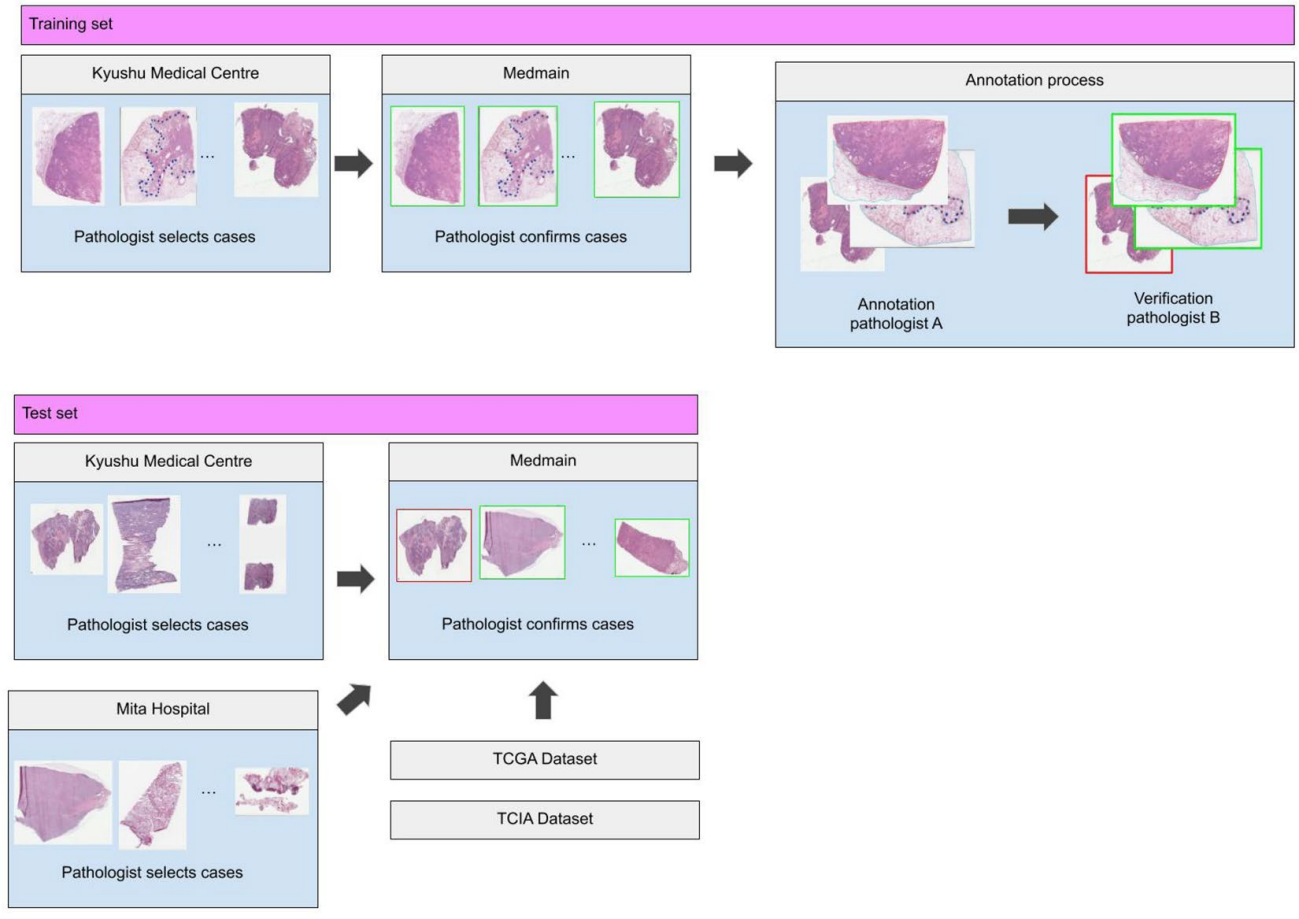
Overview of the data collection and annotation process for the training and test sets. Training data collected from Kyushu Medical Centre was first pre-selected by a pathologist and then sent over to Medmain for further filtering and selection. WSIs with concordant diagnoses were annotated by pathologists. For the test set, we collected WSIs from Kyushu medical centre, Mita hospital, and the publicly available TCGA and TCIA datasets.

The dataset obtained from Kyushu Medical Center and International University of Health and Welfare, Mita Hospital, consisted of 4,204 and 500 WSIs, respectively. Tables 2 and 3 break down the distribution of the dataset into training and test sets. The training set was solely composed of surgical images, while the test set contained some biopsy specimens (5%). All cases used for annotation were looked over carefully and verified by three pathologists prior to annotation. The WSIs were manually annotated by a group of 39 surgical pathologists (annotation pathologists) who perform routine histopathological diagnoses by drawing around the areas that corresponded to one of the two labels: carcinoma and non-neoplastic lesion. Carcinoma annotation was performed to include both carcinoma cells (tumor parenchyma) and non-neoplastic or non-viable tissue within regions of carcinoma (inflammatory cell infiltration, fibrosis, or necrosis). Figure 1 shows examples of carcinoma annotations. Several histopathological classifications of lung carcinoma exist; the one most widely used is originally proposed by Dr. Kreyberg in 1961^27^. It includes the following major categories: adenocarcinoma, squamous cell carcinoma, small cell carcinoma, large cell carcinoma, adenosquamous carcinoma, and sarcomatoid carcinoma/carcinosarcoma. The relative frequencies of the various microscopic types of lung carcinoma have changed over the years; squamous cell carcinoma used to be the more common type, however, presently, there is a majority of adenocarcinomas^28^. In this study, annotated training sets included the following major categories (subtypes): adenocarcinoma (including bronchioloalveolar carcinoma and some types of clear cell carcinoma), squamous cell carcinoma (including some types of clear cell carcinoma) and small cell carcinoma. Any large regions that did not contain carcinoma were included under the non-neoplastic labels, which consisted of cystic diseases, bronchopulmonary sequestration, bronchiectasis, abscess, granulomatous inflammation, diffuse pulmonary injury, interstitial lung disease, pneumonia, and other non-neoplastic diseases as well as normal. Non-neoplastic annotation was performed to include both parenchymal and stromal cells. Annotations performed by pathologists were modified (if necessary), confirmed, and verified by another group of two pathologists and used as training datasets. Each annotated WSI was observed by at least two pathologists, with the final checking and verification performed by a senior pathologist. Cases that had discrepancies in the annotation labels were excluded from training. Some WSIs contained multiple annotation labels. Therefore, a single WSI label of major diagnosis was assigned to a given WSI based on the following order of priority: carcinoma, non-neoplastic. For instance, if the WSI contained annotations for both carcinoma and non-neoplastic, then the WSI diagnosis was carcinoma. Figure 6 gives an overview of the collected datasets.

**Table 2:** Number of WSIs used in the training set from Kyushu Medical Centre.

	Number of WSIs
Carcinoma	1,941
Non-neoplastic	1,613
Total	3,554

**Table 3:** Summary of number of WSIs in each test set.

	Kyushu Medical Centre	Mita Hospital	TCGA	TCIA
Carcinoma	148	227	591	333
Non-neoplastic	352	273	89	167
Total	500	500	680	500

In addition to the above two datasets, we used WSIs from The Cancer Genome Atlas (TCGA) and The Cancer Imaging Archive (TCIA)^29^. We used a total of 680 WSIs from the TCGA-LUAD and TCGA-LUSC projects, and a total of 500 from the CPTAC-LSCC^30^ project.

### Deep learning models

**Figure 7:**
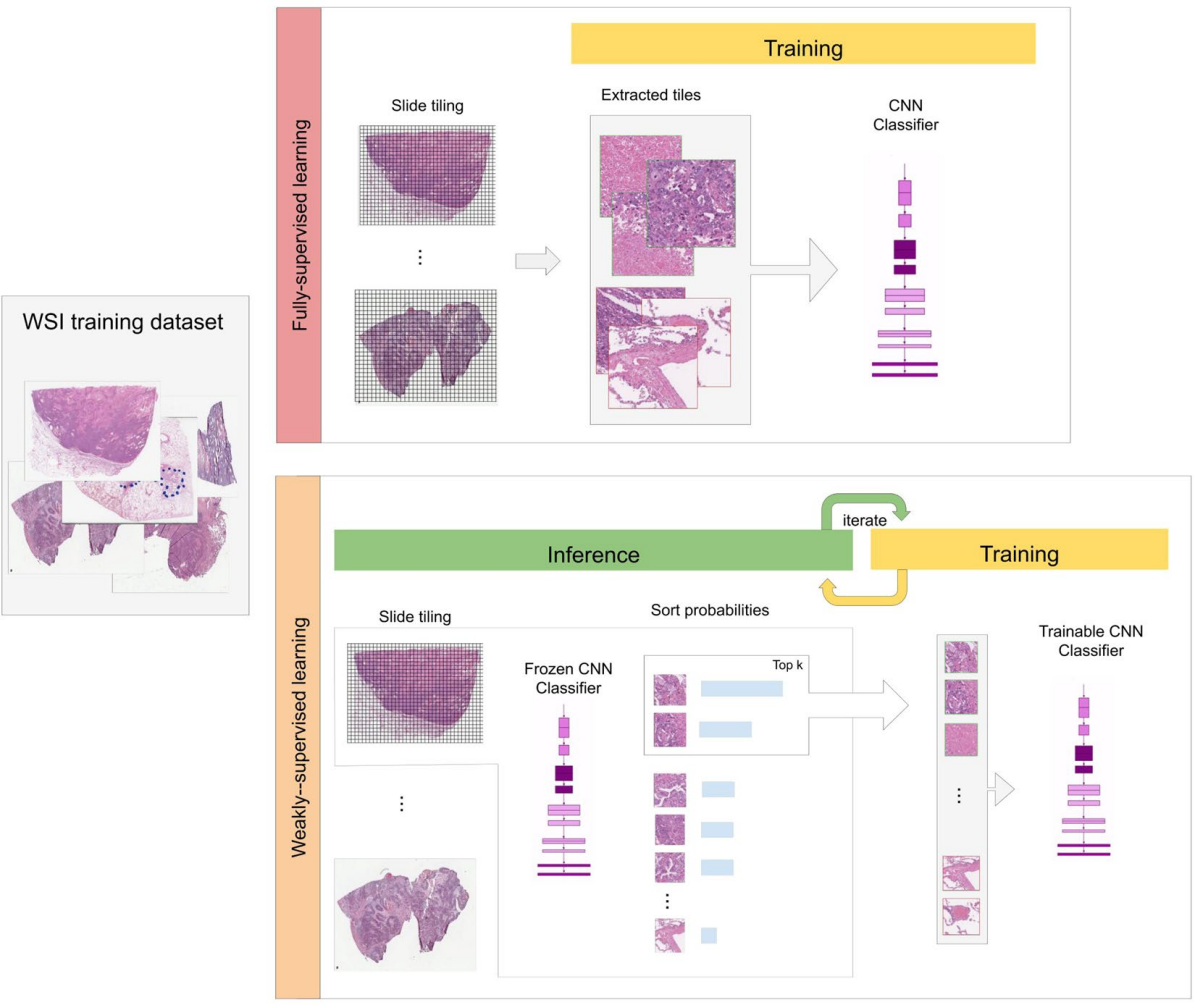
Overview of the two training methods: fully-supervised learning and weakly supervised learning. In the fully-supervised learning all the tiles from the WSIs were used for training and their labels were assigned directly based on the labels of the annotation regions they belonged to. In the weakly-supervised method, we iteratively alternated between inference and training. During inference, the model weights were frozen, and it was applied in a sliding window fashion on each WSI. The top k tiles with the highest probabilities were then selected from each WSI. During training the selected tiles were then used to train the model.

We used the EfficientNet-B3 CNN architecture^23^. We used two approaches for training: supervised learning and weakly-supervised learning to train the models. We operated on the WSIs by downsampling the magnification to x10, which was chosen as a good compromise between classification performance and speed. Figure 7 gives an overview of the two training methods.

For the supervised learning approach, the WSIs were divided into grids and tiles of size 512 × 512 pixels were extracted, resulting in about three million tiles for training. The label of the tile was assigned based on the label of the annotation region it belonged to. We then trained the CNN using the Adam optimisation algorithm^31^ with beta_1 = 0.9 and beta_2 = 0.999. We used a decaying learning rate, with a decay of 0.9 after each epoch, and with a warm start, starting from a learning rate of 0.001 up to a maximum learning rate of 0.05 during the first epoch, with an epoch being a single pass through the tiles training set. We used a batch size of 64, and the training was run for 22 epochs, with a total of about 1 M iterations. The performance of the model was tracked on a validation set. We used an early stopping approach with a patience of 10 epochs, meaning that training would stop if no improvement was observed for 10 epochs past the lowest validation loss. The model with the lowest validation loss was chosen as the final model.

For the weakly-supervised learning approach using MIL, we used an approach inspired by Campanella *et al*.^15^ with a couple of changes. We started from a pre-trained model on the ImageNet dataset, we then performed an initial phase of training on randomly sampled 512 × 512 tiles (k = 32 from each WSI) from the positively and negatively annotated regions that lasted for two epochs, with an epoch being a single sweep through the WSIs training set. After the 2nd epoch, training was switched to MIL. During a training epoch, the WSIs are iterated through in a random order. On each WSI, the model was applied in inference mode using a sliding window fashion on all its tiles, and the tiles with the top k (we used a linear decay from k = 5 to k = 1) probabilities for carcinoma were selected for training. The reason for selecting the tiles with top k probabilities is as follows: if the WSI contains carcinoma, then there should be at least one tile that contains carcinoma tissue, and, therefore, its probability should be close to one, so it should be used for training as an example of carcinoma. However, if the WSI does not contain carcinoma, then the top k tiles represent non-neoplastic tiles that have the highest probabilities for carcinoma, and, therefore, should be used for training as they are most likely to be misclassified. Once a given number of tiles has been accumulated (n = 512), a training step was performed using the Adam optimiser with a batch size of 64. We used a decaying learning rate starting from 0.001.

In both approaches, we used data augmentation in the form of tile flips, translations, and colour shifts in order increase robustness and add regularisation to the networks.

To obtain a WSI classification, the model was applied in a sliding window fashion using a stride of 256, then the WSI was assigned the label of the maximum probability of its tiles.

### Software and statistical analysis

The deep learning models were implemented and trained using TensorFlow^32^. AUCs were calculated in python using the scikit-learn package^33^ and plotted using matplotlib^34^. The 95% CIs of the AUCs were estimated using the bootstrap method^35^ with 1000 iterations.

## Data Availability

Due to specific institutional requirements governing privacy protection, the majority of datasets used in this study are not publicly available. The external lung TCGA (TCGA-LUAD and TCGA-LUSC projects) and TCIA datasets are publicly available through the Genomic Data Commons Data Portal (https://portal.gdc.cancer.gov/) and the Cancer Image Archive (https://www.cancerimagingarchive.net/), respectively.

## References

[1] Bray F (2018). Global cancer statistics 2018: Globocan estimates of incidence and mortality worldwide for 36 cancers in 185 countries. CA: a cancer journal for clinicians.

[2] Chang HY (2019). Artificial intelligence in pathology. Journal of pathology and translational medicine.

[3] Fuchs TJ, Buhmann JM (2011). Computational pathology: Challenges and promises for tissue analysis. Computerized Medical Imaging and Graphics.

[4] Hou, L. *et al*. Patch-based convolutional neural network for whole slide tissue image classification. *Proceedings of the IEEE Conference on Computer Vision and Pattern Recognition* 2424–2433 (2016). 10.1109/CVPR.2016.266 PMC5085270 27795661

[5] Madabhushi A, Lee G (2016). Image analysis and machine learning in digital pathology: Challenges and opportunities. Medical Image Analysis.

[6] Litjens G (2016). Deep learning as a tool for increased accuracy and efficiency of histopathological diagnosis. Scientific reports.

[7] Kraus OZ, Ba JL, Frey BJ (2016). Classifying and segmenting microscopy images with deep multiple instance learning. Bioinformatics.

[8] Korbar, B. *et al*. Deep learning for classification of colorectal polyps on whole-slide images. *Journal of pathology informatics* **8** (2017). 10.4103/jpi.jpi_34_17 PMC5545773 28828201

[9] Luo X (2017). Comprehensive computational pathological image analysis predicts lung cancer prognosis. Journal of Thoracic Oncology.

[10] Coudray N (2018). Classification and mutation prediction from non–small cell lung cancer histopathology images using deep learning. Nature medicine.

[11] Wei JW (2019). Pathologist-level classification of histologic patterns on resected lung adenocarcinoma slides with deep neural networks. Scientific reports.

[12] Gertych A (2019). Convolutional neural networks can accurately distinguish four histologic growth patterns of lung adenocarcinoma in digital slides. Scientific reports.

[13] Bejnordi BE (2017). Diagnostic assessment of deep learning algorithms for detection of lymph node metastases in women with breast cancer. Jama.

[14] Saltz J (2018). Spatial organization and molecular correlation of tumor-infiltrating lymphocytes using deep learning on pathology images. Cell reports.

[15] Campanella G (2019). Clinical-grade computational pathology using weakly supervised deep learning on whole slide images. Nature medicine.

[16] Iizuka O (2020). Deep learning models for histopathological classification of gastric and colonic epithelial tumours. Scientific Reports.

[17] Zhou Z-H (2018). A brief introduction to weakly supervised learning. National Science Review.

[18] Dietterich TG, Lathrop RH, Lozano-Pérez T (1997). Solving the multiple instance problem with axis-parallel rectangles. Artificial intelligence.

[19] Andrews, S., Hofmann, T. & Tsochantaridis, I. Multiple instance learning with generalized support vector machines. *Eighteenth national conference on Artificial intelligence* 943–944 (2002).

[20] Zhang, C., Platt, J. C. & Viola, P. A. Multiple instance boosting for object detection. *Advances in neural information processing systems* 1417–1424 (2006).

[21] Babenko B, Yang M-H, Belongie S (2010). Robust object tracking with online multiple instance learning. IEEE transactions on pattern analysis and machine intelligence.

[22] Sudharshan P (2019). Multiple instance learning for histopathological breast cancer image classification. Expert Systems with Applications.

[23] Tan, M. & Le, Q. Efficientnet: Rethinking model scaling for convolutional neural networks. In *International Conference on Machine Learning*, 6105–6114 (2019).

[24] Grossman RL (2016). Toward a shared vision for cancer genomic data. New England Journal of Medicine.

[25] Mitsudomi T (2010). Gefitinib versus cisplatin plus docetaxel in patients with non-small-cell lung cancer harbouring mutations of the epidermal growth factor receptor (wjtog3405): an open label, randomised phase 3 trial. The lancet oncology.

[26] Hida T (2017). Alectinib versus crizotinib in patients with alk-positive non-small-cell lung cancer (j-alex): an open-label, randomised phase 3 trial. The Lancet.

[27] Kreyberg L (1961). Main histological types of primary epithelial lung tumours. British journal of cancer.

[28] Wahbah M, Boroumand N, Castro C, El-Zeky F, Eltorky M (2007). Changing trends in the distribution of the histologic types of lung cancer: a review of 4,439 cases. Annals of diagnostic pathology.

[29] Clark K (2013). The cancer imaging archive (tcia): maintaining and operating a public information repository. Journal of digital imaging.

[30] National cancer institute clinical proteomic tumor analysis consortium (cptac). radiology data from the clinical proteomic tumor analysis consortium lung squamous cell carcinoma [cptac-lscc] collection [data set], 10.7937/k9/tcia.2018.6emub5l2 (2018).

[31] Kingma, D. P. & Ba, J. Adam: A method for stochastic optimization. In Bengio, Y. & LeCun, Y. (eds) *3rd International Conference on Learning Representations, Conference Track Proceedings* (2015).

[32] Abadi, M. *et al*. TensorFlow: Large-scale machine learning on heterogeneous systems. Software available from tensorflow.org (2015).

[33] Pedregosa F (2011). Scikit-learn: Machine learning in Python. Journal of Machine Learning Research.

[34] Hunter JD (2007). Matplotlib: A 2d graphics environment. Computing in Science & Engineering.

[35] Efron, B. & Tibshirani, R. J. *An introduction to the bootstrap* (CRC press, 1994).

[36] Timmermans WMC, Van Laar JAM, Van Hagen PM, Van Zelm MC (2016). Immunopathogenesis of granulomas in chronic autoinflammatory diseases. Clinical & translational immunology.

[37] Shah KK, Pritt BS, Alexander MP (2017). Histopathologic review of granulomatous inflammation. Journal of clinical tuberculosis and other Mycobacterial Diseases.

